# The Treatment of Pneumonia in Private Practice

**Published:** 1907-12

**Authors:** William Ewart

**Affiliations:** Senior Physician to St. George’s Hospital, and to the Belgrave Hospital for Children


### The Treatment of Pneumonia in Private Practice

By WILLIAM EWART, M. D. (Cantab), F. R. C. P.
Senior Physician to St. George's Hospital, and to the Belgrave Hospital for Children.

(*Folia Therapeutica*, October, 1907)

*The opportunity for treatment.*—In private practice pneumonia is fortunately not so fatal as in hospitals, but it remains one of the most anxious and responsible diseases to treat. For once, the hospital patient does not enjoy the better chance, as too often the delays and the exertions inseparable from his admission seriously detract from his advantages. But in private there is no acute attack which presents a more complete opportunity for treatment. *Unmistakable* in its diagnosis, it is also from the first *disabling,* and brings the case *at once* within the reach of help. Moreover, we are given ample warning of the impending pathological changes, two days on the average elapsing before the occurrence of thorough consolidation. The question, therefore, is squarely before us: Shall I treat the condition or only the circumstances?

—a question of great responsibility in all its aspects, but of paramount importance to the patient. The next question is: What treatment shall it be? The third question: When shall it be applied? This short article may help to answer these questions, for they have not yet received, even in our latest therapeutical literature, any satisfactory solution.

*The merits of the pneumococcus.*—But there are two larger questions to be dealt with first which cannot be taken for granted. Their issues are fundamental. *Does nature herself do the best* by way of treatment for the patient? And above all: Is pneu*monia an affection in itself desirable* for us to have, and to have thoroughly? This is not an idle enquiry in these days of vaccination and immunisation. Among the laity, even the educated, a belief has prevailed and has been acted upon that a variety of diseases, all of them infective, in particular mumps, scarlet fever, measles, and, alas! whooping cough, are worth inviting for the benefit of the children "that they may get it over." If the sufferers should escape the mortal risks which they are thus made to incur, it cannot be denied that something has been gained in the direction of future immunity. But in the case of pneumonia this argument does not apply. It is well known that, as in the analogous infection of influensa, the worse the pneumonic attack, so much greater is the chance that it may be *repeated* in the individual. Pneumonia cannot, therefore, be welcome. But when it does come, may it not be best to get it "sharply and thoroughly"? In short, if it were possible to mitigate the attack considerably, so as to reduce its duration, the temperature, the other dangerous symptoms, and the abundance of the micrococci, would it be better not to attempt to interfere, but to go through with the normal attack? If that point were put to the test of personal preference, opinions might differ; but it is probable that the best judges, say the average of bacteriologists, if sentenced to grow the pneumococcus in their own economy, would choose to have rather less than more of it on board, and for the shortest period. This is well worth bearing in mind when we are called in to see an unfortunate patient who has just had his pneumonic rigor.

*The pneumococcus invasion: Its mechanism and significance.*—In these matters, which are of the first importance but very obscure, there is no chance for us, though we may be entirely in error, but to speak and think in the terms of the knowledge of the day. We have, therefore, to assume that the pneumonic attack consists in an overwhelming proliferation of the microbe within the organism, and that it ends with the dying off of the microbe and with its clearance by phagocytes. We further assume that this proliferation occurs mainly *after the invasion.* On the other hand it is fairly obvious for various reasons, but above all because the patient was already ailing before, that the so-called "invasion" is not the precise moment of entrance. The initial rigor may be regarded as a medullary event, perhaps local by embolism or congestion, possibly only reflex like the ringing of an electric bell from some distant call. At any rate it cannot be the deed of a handful of pneumococci as they pass in. The significance is rather that the enemy has reached the citadel, and, unless fought, will multiply unchecked.

*The post-invasion proliferation* is still, I believe, a postulate. My friend, Dr. A. H. Smith, of New York has taught us to regard the proliferation as a *localised process,* its site the lobe affected, its nutrient *breeding ground* the inflammatory exudate in the air cells. These are inferences which seem highly probable, though still lacking proof. The inferences are drawn from the following considerations: in other pneumococcus affections, such as primary pneumococcus arthritis, or peritonitis, or meningitis, lesions are found elsewhere, but the lung may be perfectly normal. The lung is therefore presumably not in those cases the "breeding-ground," whilst everything points to the special organ which presents the inflammatory change being the seat where the microörganism multiplies. In pneumonia it would therefore be warrantable to conclude that the microörganic growth has its main seat in the lobe affected, and that its amount bears some proportion to the amount of the inflammatory exudate. It is essential to our therapeutics that we should be clear on this point: as, if these assumptions were correct, any control we might succeed in obtaining over the local inflammatory change would bear the interpretation that some degree of control has been gained over the process of proliferation itself. This is the second line of thought which should engage our attention at our first visit.

*Is nature's "self cure" adequate?*—We have no criterion: and we might argue the point for ever, as in any other "matter of opinion." But we have senses of observation, and common sense, and clinical instinct, and they speak with one voice. Why should nature's cure be the *optimum* in pneumonia when it is not a life-saving cure in erysipelas and cellulitis, in syphilis, in diphtheria, in appendicitis and in a host of infective affections capable of recovery by suitable treatment only. There is no proof, but analogy strongly disallows, that lives might not be saved by suitable interference in pneumonia. Moreover, although nature seems to do the needful in many cases that recover spontaneously, the patient's precarious survival cannot be held to prove that her method was for them the safest, the quickest, or the most free from avoidable evils. As a fact, her method consists in providing free scope, not in opposing any check, to the growth of the micrococcus. If the upshot is the loss of so many lives which might have been saved, then not to attempt to do anything is only too productive in results: and the so-called "expectant treatment" is equivalent to a sinister activity.

This is a third meditation which is urgent before we quit the bedside.

